# The pattern and mechanism of air pollution in developed coastal areas of China: From the perspective of urban agglomeration

**DOI:** 10.1371/journal.pone.0237863

**Published:** 2020-09-28

**Authors:** Yongjie Shan, Xujing Wang, Zhenbo Wang, Longwu Liang, Jiaxin Li, Jingwen Sun

**Affiliations:** 1 College of Geography Science, Shanxi Normal University, Linfen Shanxi, China; 2 Institute of Geographic Sciences and Natural Resources Research, Chinese Academy of Sciences, Beijing, China; 3 College of Resources and Environment, University of Chinese Academy of Sciences, Beijing, Chinah; 4 College of Geography and Environmental Sciences, Zhejiang Normal University, Jinhua Zhengjiang, China; Institute for Advanced Sustainability Studies, GERMANY

## Abstract

The green development of coastal urban agglomerations, which are strategic core areas of national economic growth in China, has become a major focus of both academics and government agencies. In this paper, China's coastal urban agglomeration is taken as the research area, aiming at the serious air pollution problem of coastal urban agglomeration, geographic information system (ArcGIS10.2) spatial analysis and the spatial Dubin model were applied to National Aeronautics and Space Administration atmospheric remote sensing image inversion fine particulate matter (PM_2.5_) data from 2010–2016 to reveal the temporal and spatial evolution characteristics and Influence mechanism of PM_2.5_ in China's coastal urban agglomerations, with a view to providing a reference value for coordinating air pollution in the coastal cities of the world. From 2010–2016, the PM_2.5_ concentration in China's coastal urban agglomerations decreased as a whole, and large spatial differences in PM_2.5_ concentration were observed in China's coastal urban agglomerations; the core high-pollution areas were the Beijing–Tianjin–Hebei, Shandong Peninsula, and Yangtze River Delta urban agglomerations. Large spatial differences in PM_2.5_ concentration were also observed within individual urban agglomerations, with higher PM_2.5_ concentrations found in the northern parts of the urban agglomerations. Significant spatial autocorrelation and spatial heterogeneity were observed among PM_2.5_-polluted cities in China's coastal urban agglomerations. The northern coastal urban agglomerations formed a relatively stable and continuous high-pollution zone. The spatial Dubin model was used to analyze the driving factors of PM_2.5_ pollution in coastal urban agglomerations. Together, meteorological, socioeconomic, pollution source, and ecological factors affected the spatial characteristics of PM_2.5_ pollution during the study period, and the overall effect was a mixed effect with significant spatial variation. Among them, meteorological factors were the greatest driver of PM_2.5_ pollution. In the short term, the rapid increase in population density, industrial emissions, industrial energy consumption, and total traffic emissions were the important driving factors of PM_2.5_ pollution in the coastal urban agglomerations of China.

## 1 Introduction

Coastal urban agglomerations are at the heart of the global urbanization process, and as the highest form of urban development, the formation and rapid expansion of such urban agglomerations, where the population is highly concentrated [1], have put tremendous pressure on the ecological environment [2]. Therefore, the green development of coastal urban agglomerations have become a focus on scholars. Since 1978, China is experiencing rapid urbanization and severe ecological and environmental challenges, and within China, the coastal areas have experienced the fastest economic development [3]. The coastal area as a key area for population migration and economic development [4], high-intensity development has resulted in serious ecological and environmental problems in coastal urban agglomerations, with PM_2.5_ pollution being particularly serious. Coastal urban agglomerations have become highly polluted with fine particulate matter (PM_2.5_). In 2018, more than 60% of the 101 cities in China's coastal cities had smog orange warnings. However, these cities are not balanced in terms of their spatial distribution; they are mainly concentrated in large-scale urban agglomerations in the middle and high latitudes (e.g., the Beijing–Tianjin–Hebei and Yangtze River Delta urban agglomerations). In addition, large differences are found in the time series and sources of PM_2.5_ contamination. Although many scholars have discussed the temporal and spatial patterns of PM_2.5_ pollution at the global [5], national [6], urban agglomeration [7], and urban [8] levels, few studies have examined the temporal and spatial evolution characteristics and driving factors of PM_2.5_ pollution in China's coastal urban agglomerations.

Past studies have primarily been based on two types of research data: remote sensing image retrieval of atmospheric aerosol thickness (AOD) and real-time monitoring point data. Existing studies mainly used the energy-dispersive X-ray spectroscopy model [9] Backward air parcel trajectories method [10], land to use regression [11], mixed regression [12], and spatial metrology models [13] to evaluate the chemical characteristics [14], spatial heterogeneity [15], pollution sources [16], human health risks [17], and influencing factors [18] of PM_2.5_ pollution. Numerous studies have shown that meteorological elements play a key role in the air pollution PM_2.5_ concentration [19]. Meanwhile, natural and socioeconomic factors such as topographic conditions [20], transportation [21], high density construction [22], air speed reduction [23], and biomass burning [24] have important effects on the generation and transmission of atmospheric pollution. Natural external factors have mainly negative effects on PM_2.5_ pollution, while social and economic factors have primarily positive effects [25]. Some scholars have proposed prevention and control strategies for PM_2.5_ pollution, including hierarchical, cross-regional, and multidirectional air pollution control models [26], multiagent collaborative governance systems [27], and meteorological science and technology upgrades [28].

Overall, there is a lack of literature comparing PM_2.5_ pollution in different urban agglomerations and characterizing PM_2.5_ pollution in coastal urban agglomerations. The rapid economic and social development and the associated air pollution in highly developed coastal urban agglomerations have major effects on China's sustainable development, global ecological and environmental security, and climate change. Thus, it is necessary to explore PM_2.5_ pollution in China’s coastal urban agglomerations. Understanding the temporal and spatial evolution characteristics along with the main driving factors of PM_2.5_ pollution in these regions will provide theoretical support for sustainable development and climate change research. In this study, remote sensing imagery was used to retrieve AOD data and visualize the PM_2.5_ concentrations of China's coastal urban agglomerations to reveal the temporal and spatial variations in PM_2.5_ pollution. In addition, the spatial Dubin model was applied to reveal the factors influencing PM_2.5_ pollution in urban agglomerations. Finally, the results were used to reveal differences, identify problems, and propose corresponding countermeasures.

## 2 Study area and methodology

### 2.1 Research area

**Fig 1:**
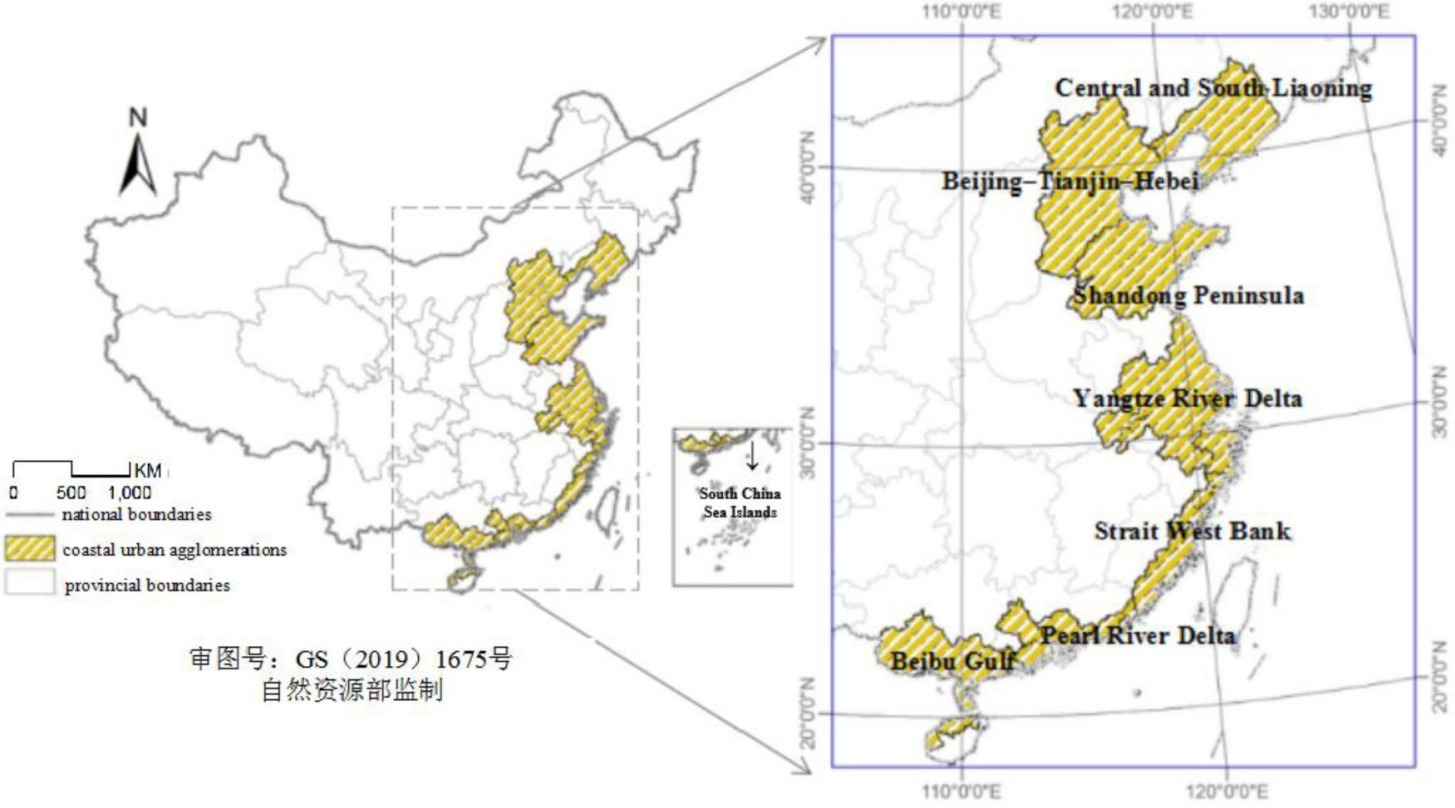
Maps showing the study area, which included seven major urban agglomerations in China.

China’s “National New Urbanization Planning (2014~2020)” proposes 19 major urban agglomerations [29]. This study focused on seven coastal urban agglomerations: Central and South Liaoning, Beijing–Tianjin–Hebei, Shandong Peninsula, Yangtze River Delta, Strait West Bank, Pearl River Delta, and Beibu Gulf (Fig 1). Three national-level Urban Agglomerations (Beijing–Tianjin–Hebei, Yangtze River Delta, Pearl River Delta), while four are regional-level urban agglomerations (Central and South Liaoning, Shandong Peninsula, Strait West Bank, and Beibu Gulf). The total area of cities in China are approximately 10.78% of the total area of the country. In 2018, the total population in cities were 550 million, accounting for 40.2% of the national population; the GDP produced by urban areas in 2018 was 50.57 trillion dollars, accounting for 56.17% of the national GDP (Table 1). This paper discusses the spatial and temporal distribution characteristics of PM_2.5_ concentration in coastal urban agglomerations from 2010 to 2016 on the city scale and analyzes the main factors contributing to PM_2.5_ pollution in different urban agglomerations.

**Table 1:** Summary of the area, population, and contribution to GDP for China's coastal urban agglomerations in 2017.

Urban Agglomeration	Area (km^2^)	Population (ten thousands)	GDP (trillion dollars)
Central and South Liaoning	21.2	3075	2.4
Beijing–Tianjin–Hebei	9.71	11000	8.5
Shandong Peninsula	27.0	10047	7.6
Yangtze River Delta	21.5	15000	17.8
Strait West Bank	5.5	5729	4.2
Pearl River Delta	7.3	6300	8.1
Beibu Gulf	11.7	3935	1.9

### 2.2 Construction of the factor system and data sources

#### 2.2.1 Construction of the factor system

**Fig 2:**
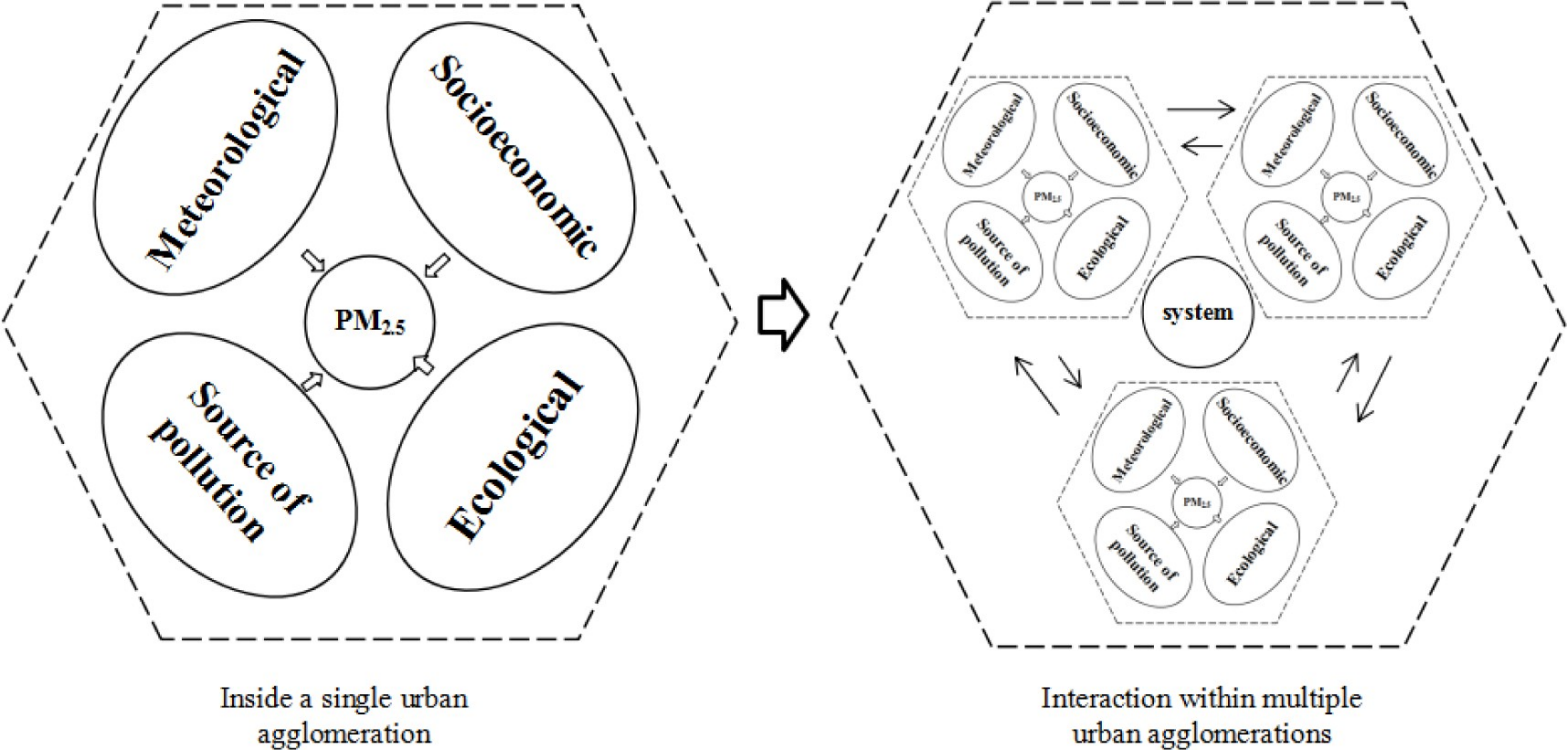
Interaction between factors affecting PM_2.5_ pollution within individual urban agglomerations and between multiple urban agglomerations.

The factors affecting PM_2.5_ pollution in China's coastal urban agglomerations are complex and diverse. In addition to natural conditions, PM_2.5_ pollution is closely related to human factors such as industrial production, tail gas emissions, and coal combustion. This study considered the pollution source, meteorological, socioeconomic, and ecological factors affecting PM_2.5_ pollution. In total, 17 indicators of influencing potential influencing factors (Table 2, Fig 2). The pollution sources are related to the formation of PM_2.5_ pollution and include automobile exhaust (TE) [30], building dust (Bd) [9], industrial emissions (IE), and industrial energy consumption (IEC) [31]. Meteorological elements refer to the natural factors that affect PM_2.5_ pollution, including annual average temperature (AT) [32], annual average precipitation (Pr) [33], annual average wind speed (WS) [34], and sunshine hours (S) [35]. Socioeconomic factors are related to economic and social development and include industrial agglomeration (Ia), The proportion of tertiary industry (Iha) [36], technology investment (Ti), degree of openness (Fo), population density (Pd), urbanization rate (UR), and per capital GDP (PGDP). Ecological factors include ecological safety (ES) [37], ecological cleanliness (Er) [38], and ecological damage (ER).

**Table 2:** Factors affecting PM_2.5_ concentration in coastal urban agglomerations considered in this study.

Factor type	Factor	Description	Unit
Pollution source	Industrial energy consumption (IEC)	Industrial electricity consumption	Gigawatt-hour
	Industrial emissions (IE)	Industrial smoke dust emissions	10,000 tons
	Automobile exhaust (TE)	Year-end private car + bus + total taxi	Ten thousand vehicles
	Building dust (Bd)	Built area growth rate	%
Meteorological	Annual average wind speed (WS)	Annual average of each meteorological factor	m/s
	Sunshine hours (S)		h
	Annual average temperature (AT)		°C
	Annual average precipitation (Pr)		mm
Socioeconomic	Urbanization rate (UR)	Population urbanization	%
	Per capita GDP (PGDP)	GDP/total population	Ten thousand yuan
	Population density (Pd)	Population per unit area	Person// Km^2^
	Industrial agglomeration (Ia)	Output per unit of industrial area	Ten thousand yuan/m^3^
	The proportion of tertiary industry (Iha)	Tri-production ratio	%
	Technology investment (Ti)	Technology expenditure/GDP	%
	Degree of openness (Fo)	Foreign investment/GDP	%
Ecological	Cleanliness (Er)	Industrial smoke and dust disposal capacity	10,000 tons
	Ecological safety (ES)	Urban greening rate	%
	Ecological damage (ER)	Ecological space land area reduction ratio	%

#### 2.2.2 Data sources

The 2010–2016 PM_2.5_ data for coastal cities were obtained from the National Aeronautics and Space Administration Socioeconomic Data (http://fizz.phys.dal.ca/~atmos/martin/?page_id=140)) and Applications Center. Remote sensing inversion was used to obtain 1000-m raster data. The urban administrative boundary vector data were derived from the basic geographic information provided by the National Basic Geographic Information Center of China (http://www.ngcc.cn/ngcc/). The raster data were extracted by using the study area vector boundary as a mask. The city-level administrative division lines vector data were used to calculate the annual average PM_2.5_ concentration of each city, thus establishing the PM_2.5_ concentration spatiotemporal database of the coastal urban agglomerations. All meteorological data are from China Meteorological Data Network (http://data.cma.cn/site) The original data is station data. Due to the uneven spatial distribution of monitoring points, in order to obtain the average value of PM_2.5_ concentration, Kriging interpolation method is used to obtain grid data onto 7 coastal city groups, so as to obtain the smooth PM_2.5_ concentration value, and calculate the annual average value of meteorological elements of each city. Kriging interpolation not only has the function of surface prediction, but also has high reliability. The socioeconomic data were obtained from the China City Statistical Yearbook (2011–2017), the China Regional Statistical Yearbook (2011–2017), and the statistical yearbooks of various cities (http://www.stats.gov.cn/).

## 3 Research methods

### 3.1 Spatial autocorrelation test

#### 3.1.1 Global spatial autocorrelation

According to Tobler's first law of geography, the closer a geographical thing is in space, the greater the correlation between its attribute values (i.e., the stronger the spatial dependence) [39]. Research has revealed a spatial dependence of PM_2.5_ pollution [40]. In this study, the global Moran's index (*I*) was used to test the global spatial autocorrelation of PM_2.5_ concentration. *I* was calculated as follows:

$$I=\frac{n}{S_{0}}*\frac{\sum_{i=1}^{n}\sum_{j=1}^{n}w_{ij}z_{i}z_{j}}{\sum_{i=1}^{n}z_{i}^{2}}$$ (1)

$$S_{0}=\sum_{i=1}^{n}\sum_{j=1}^{n}w_{ij};\;Z_{i}=Y_{i}‐\bar{Y};\;Z_{j}=Y_{j}‐\bar{Y}$$ (2)

In the above formula, *n* is the number of regions, and *W*_*ij*_ is the spatial weight matrix. Generally, the adjacent cells are taken as 1 and the others as 0. *Y*_*i*_ is the PM_2.5_ concentration value of the *i* region, *Y*_*j*_ is the PM_2.5_ concentration value of the *j* region, and $\bar{Y}$ is the average PM_2.5_ concentration of the region. Moran's I, *I* takes a value between [−1,1]. When *I* < 0, there is a negative correlation between the regional units; when *I* = 0, there is no correlation between the regional units; and when *I* > 0, there is a positive correlation between the regional units. The closer *I* is to 1, the closer the relationship between the regional unit attribute values; the closer *I* is to 0, the more unrelated the attribute values between the regional units; the closer *I* is to −1, the greater the difference in attribute values between the units.

#### 3.1.2 Local spatial autocorrelation

Local autocorrelation analysis can be used to measure the degree of influence of a local spatial unit relative to the overall study area (e.g., the correlation between the air quality of a regional unit and the air quality characteristics of adjacent units). The local Moran’s index *I*_*i*_ is calculated as

$$\begin{array}{cc} & \begin{array}{cc} & I_{i}=\frac{n\left(x_{i}-\bar{x}\right)\sum_{j=1}^{m}W_{ij}\left(x_{j}-\bar{x}\right)}{\sum_{i=1}^{n}{\left(x_{i}-\bar{x}\right)}^{2}}\left(i\neq j\right) \end{array} \end{array},$$ (3)

where *x_i_* and *x_j_* are the air quality observations of cities *i* and *j*, respectively; *n* is the number of cities; *W*_*ij*_ is the space weight; and *i* = 1,2,…,*n* and *j* = 1,2,…,*m*, where *m* is the number of cities geographically adjacent to city *i*. The academic community usually uses the standardized statistic *Z* to test whether the Moran index has spatial autocorrelation. The *Z* value for the local Moran’s index, *Z*_*i*_, is given by

$$Z\left(I\right)=\frac{I-E[I]}{\sqrt{V[I]}}$$ (4)

where:

$$E[I]=-1/(n-1),\;V[I]=E[I^{2}]-E{\left[I\right]}^{2}$$ (5)

Where: it can measure the significance level of Moran's I index, which is the expectation of concentration autocorrelation of PM_2.5_ and the coefficient of variation. In this study, the significance level was set at 0.01. At this level, when |*Z*(*I*)|<2.58, the spatial autocorrelation of PM_2.5_ concentration is not significant (i.e., PM_2.5_ concentration shows an independent random distribution). When *Z*(*I*) < −2.58, PM_2.5_ concentration shows negative spatial autocorrelation. It showed that PM_2.5_ concentration had a negative correlation in spatial distribution, and its attribute values showed a dispersive distribution, i.e. high-low correlation of high PM_2.5_ concentration unit surrounded by low PM_2.5_ concentration unit and low-high correlation of low PM_2.5_ concentration unit surrounded by high PM_2.5_ concentration unit… When *Z*(*I*) > 2.58, It shows that PM_2.5_ concentration has a positive autocorrelation in spatial distribution, If the concentration of PM_2.5_ in this unit and its adjacent units is higher than the average, it is called "hot spot"; if the concentration of PM_2.5_ in this unit and its adjacent units is lower than the average, it is called "cold spot".

### 3.2 Construction of the spatial measurement model

The core thought based on the spatial difference of geography, the spatial econometric model is included in the spatial weight matrix, considering the spatial correlation between the elements, which is closer to the objective law than the classical econometric model. As a regional characteristic, urban PM_2.5_ pollution is not an independent measurement; adjacent areas can affect the trends in PM_2.5_ pollution, and strong spatial spillover can occur. Thus, when analyzing the driving force of PM_2.5_ pollution in urban agglomerations, the spatial effect cannot be ignored and should be estimated using a spatial econometric model. From the perspective of the data structure, the spatial measurement model is primarily divided into the spatial section measurement model and the spatial panel measurement model. Spatial cross-sectional econometric model only estimates one year's data, ignoring the time lag effect of factors. Spatial panel data model makes full use of data information, so it is more accurate to analyze the driving factors of PM_2.5_ pollution in a long period of time [41]. The commonly used spatial panel econometric models include spatial lag model, spatial error model and spatial Durbin model. The expressions of the three models are as follows:

$$Panel-SAR:\;lnPM_{it}=\alpha WlnPM_{it}+\varphi lnPM_{it-1}+\beta_{0}+\beta_{i}X_{it}+a_{i}+\gamma_{t}+\mu_{it},$$ (6)

$$Panel-SEM:\;lnPM_{it}=\beta_{0}+\beta_{i}X_{it}+\theta WZ_{it}+a_{i}+\gamma_{t}+\lambda W\nu_{it}+\mu_{it},$$ (7)

and

$$Panel-SDM:\;lnPM_{it}=\alpha WlnPM_{it}+\varphi lnPM_{it-1}+\beta_{0}+\beta_{i}X_{it}+\theta WZ_{it}+a_{i}+\gamma_{t}+\mu_{it}.$$ (8)

In Eqs (6)–(8), lnPM_it_, lnPM_it−1_, and W lnPM_it_ are the logarithm of PM_2.5_ concentration in urban areas and its post-time and spatial lag terms, respectively; WZ_it_ is the spatial lag term of explanatory variables; and a_i_, γ_t_, and μ_it_ represent individual effects, time effects, and error terms, respectively.

The spatial lag model can show that the change in PM_2.5_ in the study region is not only a local independent variable, but also a function of the changes in PM_2.5_ in other spatially associated regions; in other words, changes in PM_2.5_ have spatial spillover effects. The spatial error model fully reflects the effect of changes from PM_2.5_ pollution in the region on random disturbances that are not included in the model, while the spatial Dubin model fully considers the effects of PM_2.5_ pollution that are not reflected on the model [42]. As the standard starting point for a spatial econometric model, the spatial Dubin model is a standard framework for capturing various spatial spillover effects. The spatial Dubin model can be transformed into a common spatial lag model and a spatial error model under different coefficient settings. For generality, the spatial Dubin model was applied in this study [43]. The adjacent spatial weight matrix was used to construct the weight matrix; thus, the adjacent spatial units had significant mutual influence, and the non-adjacent spatial units had no mutual influence. The code used in the spatial Dubin model comes from Elhorst's Spatial Metrology Economic Matlab Toolbox.

## 4 Results and discussion

### 4.1 Temporal and spatial distribution characteristics of PM_2.5_ in China's coastal urban agglomerations

#### 4.1.1 Time series analysis

**Fig 3:**
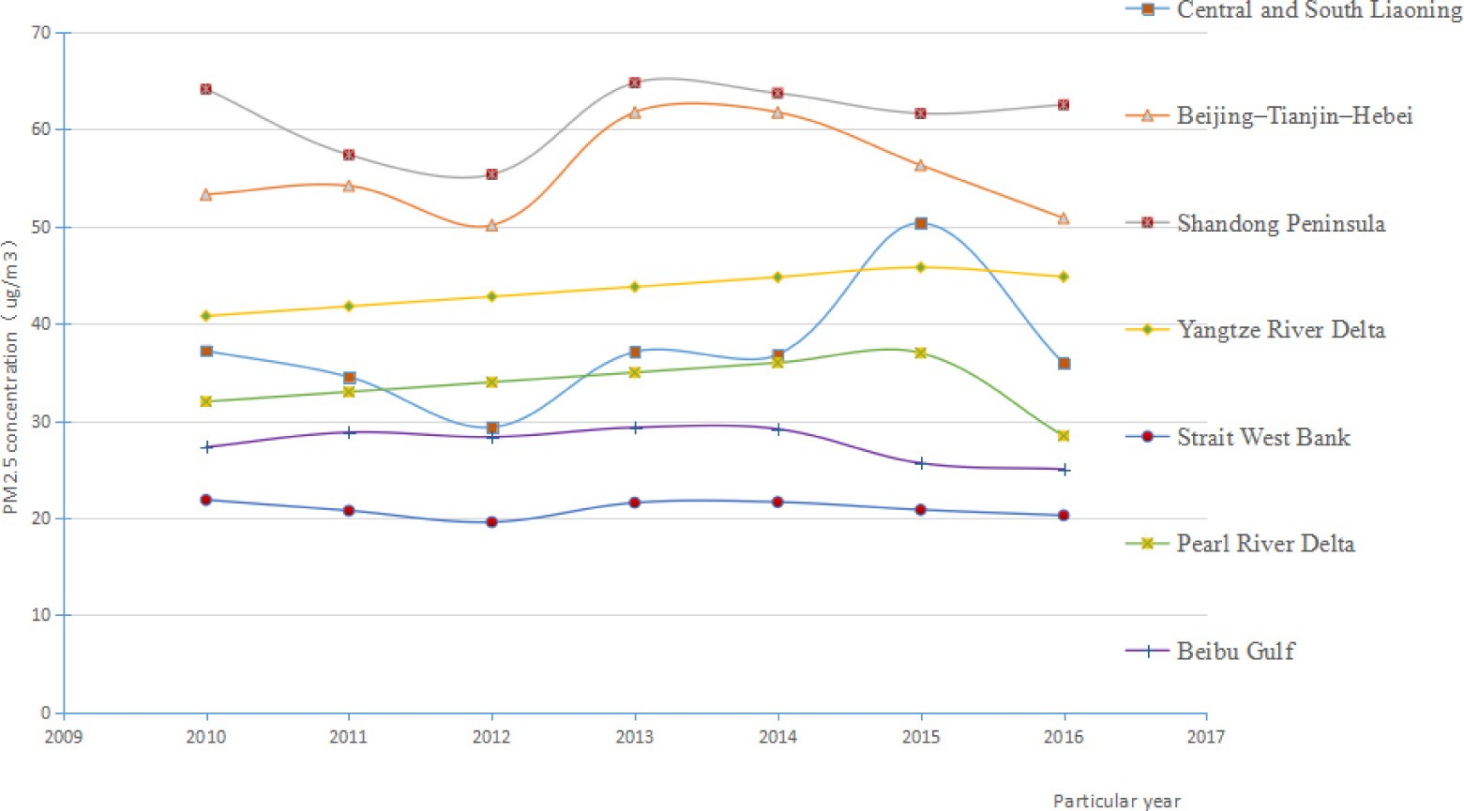
Time series of PM_2.5_ concentration for seven coastal cities in China from 2010–2016.

In this paper, the mean value of PM_2.5_ concentration of each city within the urban agglomeration are used as the PM_2.5_ concentration of the urban agglomeration, statistical analysis shows that among the seven major urban agglomerations in China's coastal areas, the PM_2.5_ concentration was highest in the Shandong Peninsula urban agglomeration followed by the Beijing–Tianjin–Hebei urban agglomeration. The air quality was the highest in the Strait West Bank urban agglomeration. From 2010–2016, the PM_2.5_ concentrations in all the urban agglomerations except the Yangtze River Delta showed downward trends (Fig 3). Among the urban agglomerations, the decrease in PM_2.5_ concentration was the most significant in the Pearl River Delta (decrease of 11.04%). The year 2013 was an important turning point in PM_2.5_ concentration in the Beijing–Tianjin–Hebei, Shandong Peninsula, Pearl River Delta, and Beibu Gulf urban agglomerations. After 2013, the air quality in the coastal urban agglomerations improved overall. From 2010 to 2016, the PM_2.5_ concentration in the different urban agglomerations decreased in the following order: Shandong Peninsula > Beijing–Tianjin–Hebei > Yangtze River Delta > Central and South Liaoning > Pearl River Delta > Beibu Gulf > Strait West Bank. Notably, the order of PM_2.5_ concentration in the urban agglomerations in Central and South Liaoning changed greatly during the study period. Accordingly, the State Council has adopted the "PM_2.5_ concentration Prevention and Control Action Plan" to formulate regional PM_2.5_ concentration target values and implement pollution prevention and control actions. The PM_2.5_ concentration in the heavily polluted Beijing–Tianjin–Hebei, Shandong Peninsula, and Central-South Liaoning urban agglomerations fluctuated in a large range with overall downward trends. The PM_2.5_ concentration in the other urban agglomerations remained relatively stable with small overall declines. The limit for annual average PM_2.5_ concentration specified in the Ambient Air Quality Standard is 35.00 μg/m^3^(GB3095-2012). During the study period, the annual average PM_2.5_ concentrations in the Beijing–Tianjin–Hebei, Shandong Peninsula, and Yangtze River Delta urban agglomerations exceeded this value. The annual average PM_2.5_ concentrations in the Strait West Bank and Beibu Gulf urban agglomerations were below the limit. For the urban agglomerations in central and southern Liaoning, the annual average PM_2.5_ concentrations were below the limit in 2011 and 2012 but above the limit in the other years. In the Pearl River Delta urban agglomeration, the annual average PM_2.5_ concentration exceeded the limit in 2014 and 2015 but was below the limit in the other years.

#### 4.1.2 Analysis of spatial patterns

**Fig 4:**
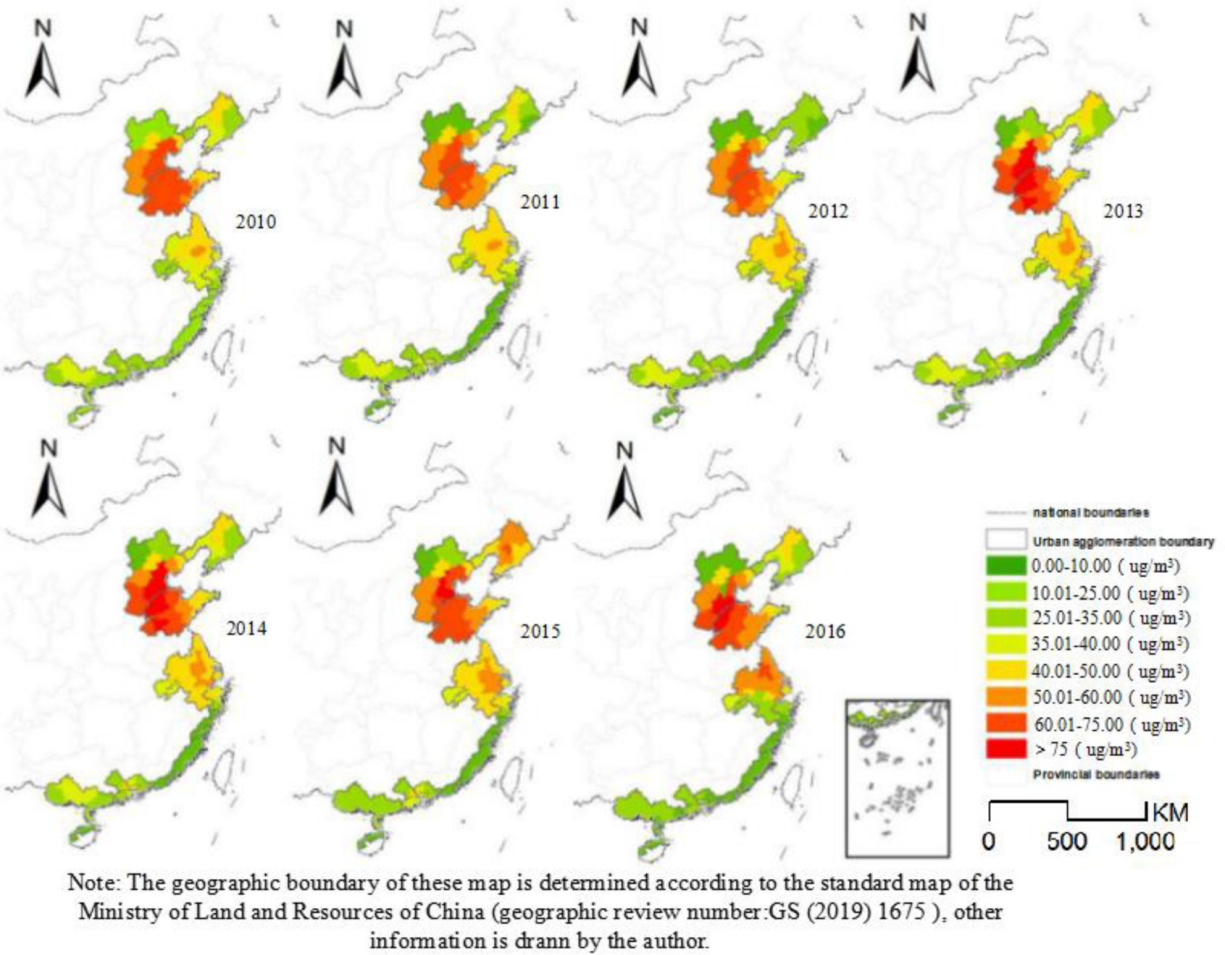
Spatial patterns of PM_2.5_ concentration in China's coastal urban agglomerations from 2010 to 2016.

Based on the annual average PM_2.5_ concentration limit of 35 μg/m^3^, the study area was divided into areas of high-concentration pollution (annual average PM_2.5_ concentration above the limit) and low-concentration pollution (annual average PM_2.5_ concentration below the limit). From 2010–2016, the overall air quality of China's coastal urban agglomerations improved (Fig 4). The number of cities with annual average PM_2.5_ concentrations below the limit increased by 10.8%, with the most significant decrease in pollution observed in the Yangtze River Delta urban agglomeration. The number of cities in low-value areas increased by 15%, and the number of cities in low-value areas of Beijing-Tianjin-Hebei urban agglomeration increased by 8%., indicating PM_2.5_ pollution remediation in China has been remarkably successful in recent years.

In terms of spatial differences, the PM_2.5_ concentrations in China’s seven coastal cities were significantly higher at high latitudes compared to at low latitudes. The Beijing–Tianjin–Hebei, Shandong Peninsula, and Yangtze River Delta urban agglomerations formed a core area of high pollution containing all the cities with annual average PM_2.5_ concentrations greater than 75 μg/m^3^. The Strait West Bank, Pearl River Delta, and Beibu Gulf urban agglomerations were areas of low PM_2.5_ concentration. Large differences in the air quality was also observed within each urban agglomeration, with higher PM_2.5_ concentrations in the north compared to in the south. The Beijing–Tianjin–Hebei urban agglomeration had the largest spatial difference. From the perspective of the internal differences of urban agglomerations, the internal differences of urban agglomerations with more serious air pollution are large, mainly Beijing Tianjin Hebei, Shandong Peninsula and the Yangtze River Delta urban agglomerations. The difference of PM_2.5_ concentration in Beijing Tianjin Hebei Urban Agglomeration shows the spatial distribution pattern of high in southeast, high in Northwest and low in Northwest. The concentration distribution pattern of Shandong Peninsula urban agglomeration is low in the East and high in the middle and West. The urban agglomeration in the Yangtze River Delta is characterized by obvious core edge pattern, with the central area as the core area of high pollution spreading to the surrounding areas. From 2010–2016, the spatial differences in PM_2.5_ concentration in the Pearl River Delta and Beibu Gulf urban agglomerations decreased, while those within the other urban agglomerations gradually increased. In terms of the spatial changes in PM_2.5_ concentration in the high-pollution urban agglomerations, decreasing trends were observed in the northern urban agglomerations (e.g., Beijing–Tianjin–Hebei), while increases were found in the southern urban agglomerations (e.g., Yangtze River Delta). This result is the law on the prevention and control of air pollution clearly stipulates that all prefecture level cities whose air quality fails to meet the national secondary standard shall timely carry out the preparation and publicity of their city's plan for meeting the atmospheric environment quality standards within a time limit, and establish a positive schedule for meeting the standards and corresponding measures. The pollution concentration base of the northern coastal city group is higher, so the decrease of concentration control is higher than that of the southern city group.

### 4.2 Analysis of PM_2.5_ spatial autocorrelation in coastal urban agglomerations

#### 4.2.1 Analysis of spatial autocorrelation index

The spatial autocorrelation of average annual PM_2.5_ concentration in the seven major coastal urban agglomerations from 2010 to 2016 was evaluated using ArcGIS software (Table 3). The global Moran’s index *I* was positive and significant at the 1% level, indicating that PM_2.5_ concentration showed significant spatial autocorrelation during the study period.

**Table 3:** Spatial autocorrelation indices for annual average PM_2.5_ concentration in China's coastal urban agglomerations from 2010 to 2016.

Particular year	2010	2011	2012	2013	2014	2015	2016
Central and South Liaoning	0.10	0.15*	0.20**	0.18**	0.12*	0.13*	0.14*
Beijing–Tianjin–Hebei	0.33**	0.33**	0.34**	0.35**	0.35**	0.34**	0.33**
Shandong Peninsula	0.58***	0.53***	0.53***	0.52***	0.53***	0.47***	0.51**
Yangtze River Delta	0.66***	0.68***	0.67***	0.67***	0.67***	0.68***	0.67**
Strait West Bank	0.11*	0.10*	0.17	0.11*	0.13*	0.21	0.20*
Pearl River Delta	0.17*	0.12*	0.14*	0.17*	0.25*	0.23*	0.21*
Beibu Gulf	0.96***	0.96***	0.97***	0.92***	0.93***	0.90***	0.89**

* represents significance at the 10% level

** represents significance at the 5% level; and

*** represents significance at the 1% level.

#### 4.2.2 Analysis of the trend of agglomeration

**Fig 5:**
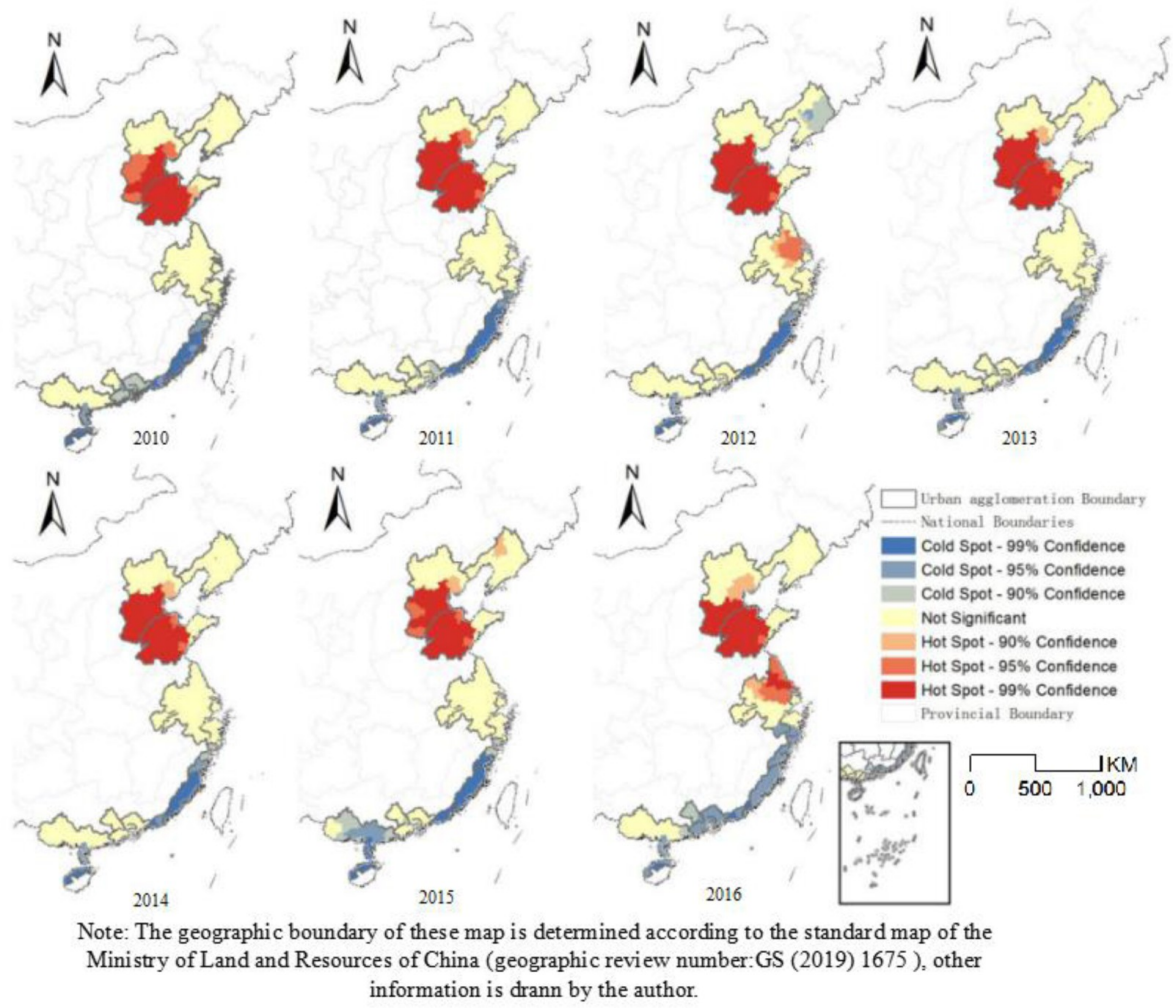
Spatial patterns of cold spots and hot spots of PM_2.5_ concentration in China's coastal urban agglomerations from 2010–2016.

From 2010–2016, the spatial distributions of hot spot and cold spot in China's coastal urban agglomerations were quite different (Fig 5). The hotspots were concentrated in the Beijing–Tianjin–Hebei and Shandong Peninsula urban agglomerations, while the cold spot were concentrated in the Strait West Bank, Pearl River Delta, and Beibu Gulf urban agglomerations. As shown in Fig 4, a relatively stable and continuous zone of high PM_2.5_ pollution was observed in the northern coastal urban agglomerations, while the southern urban agglomerations formed a stable area of good air quality. The number of cities in the pollution hot spot, which included the northern coastal urban agglomerations, decreased during the study period, while the number of cities in the southern cold spot increased. This reflects an overall improvement in air quality, indicating that environmental measures such as the PM_2.5_ concentration Prevention Action Plan have alleviated PM_2.5_ pollution.

The spatial distribution of cold spot and hot spot in PM_2.5_ concentration changed greatly at the national level, while the regional-level changes were small. Among the urban agglomerations, the number of hot spots in the Beijing–Tianjin–Hebei urban agglomeration decreased the most, reflecting the implementation of the PM_2.5_ concentration Prevention and Control Action Plan in the Beijing–Tianjin–Hebei urban agglomeration and surrounding areas. In contrast, the hot spots in the Yangtze River Delta urban agglomeration increased significantly during the study period because of the increased production capacity of traditional high-energy-consuming industries (e.g., the steel industry) in this area. From 2000 to 2016, the output of cement, steel, chemical fiber, and ethylene respectively increased by 224%, 512%, 834%, and 388% in this region. The coldspot area in the Pearl River Delta urban agglomeration increased significantly during the study period, primarily due to the implementation of the Clean Air Action Plan in 2015 along with other clean air operations.

### 4.3 Analysis of factors affecting PM_2.5_ concentration in China's coastal urban agglomerations

Overall, the *R*^2^ values for the spatial Dubin model indicated a good overall fit to the data, and the WInPM_it_ values in China's coastal urban agglomerations were significantly below 1%, indicating strong spatial endogenous interactions among PM_2.5_ pollution in China's coastal urban agglomerations. Therefore, the results of the SDM model are provided in Table 4. The degrees of influence of the different explanatory variables were compared according to the saliency and elasticity coefficient of each element (Table 4).

**Table 4:** Results from the SDM model of PM_2.5_ pollution in China's coastal urban agglomerations from 2010–2016.

Parameter	Central and South Liaoning	Beijing–Tianjin–Hebei	Shandong Peninsula	Yangtze River Delta	Strait West Bank	Pearl River Delta	Beibu Gulf
Intercept	-0.283	-3.362	6.193	21.589	-4.043	-7.733	-4.807
lnIa	0.206***	0.068	-0.048**	0.067	0.005**	0.056***	0.175**
lnIha	-0.086	-0.846***	-0.058	0.001	-0.019	-0.147***	-0.047
Ti	-0.084	0.260	0.070	-0.018	0.094***	-0.060***	-0.003
Fo	-0.491***	-0.046	-0.068	-0.053	0.373	0.038	-0.380**
lnPd	0.117***	0.357**	0.192***	-0.063	-0.001***	-0.104***	-0.718***
UR	0.532***	-0.114	0.147***	-0.012	0.229***	0.055	0.251
lnPGDP	0.342***	-0.320**	0.050**	0.073	0.047***	0.010	-0.109
lnTE	0.014	0.492***	0.037**	0.027*	0.060	0.032	0.155**
lnIEC	0.015	0.098***	-0.009	0.015*	0.007	-0.003	-0.009
lnBd	0.066**	0.051	-0.012	-0.003	-0.001	0.034**	-0.003
lnIE	0.072	0.285*	0.017	0.060	-0.001	-0.008	0.105**
lnAT	-0.386	-1.081***	0.154	1.004	-4.997***	-1.761***	-5.792***
lnPr	0.041	2.000**	0.330***	0.295	0.785***	-0.457***	-0.654
lnWS	0.126	-5.332***	-0.085	-5.428***	1.191	-1.248***	-2.922*
lnS	-7.284**	-2.662*	0.324	0.264	-0.396	-1.306	0.929*
lnEr	0.008	-0.015*	0.005	-0.009*	-0.004	-0.002*	0.014
lnES	-0.024	0.133	0.012	-0.092**	-0.086	0.001	-0.030
lnER	0.187	0.073	0.213	0.012*	0.021*	0.031**	0.037
W*lnPM_2.5_	0.397***	-0.480**	0.652***	-0.236*	0.359***	0.527***	-0.236
W*lnIa	0.048	-0.180	0.232***	0.267*	-0.008	-0.249***	-0.352**
W*lnIha	0.465***	-0.505	0.133	-0.152*	0.045***	-0.131	0.005
W*Ti	0.696	-2.415***	-0.327**	-0.223	0.299**	-0.240***	1.079**
W*Fo	0.437	4.754***	0.404***	0.782***	-0.052	0.319***	2.041***
W*lnPd	-0.141***	-0.295	-0.324***	-0.819***	-0.146**	-0.655***	1.472***
W*UR	-0.812	0.642	-0.254**	-0.459	-0.132*	-0.518***	-0.281
W*lnPGDP	0.127	0.133	-0.280***	-0.150	-0.240	0.116	-0.154
W*lnTE	-0.091	2.325***	-0.005	-0.280	0.116**	-0.085	-0.222
W*lnIEC	-0.013	0.238***	-0.026	0.019	0.042***	0.005	0.028
W*lnBd	0.167*	0.254	0.069***	0.054	0.005	-0.028	-0.068
W*lnIE	-0.099	-0.444	-0.016	0.275**	0.003	0.014	-0.133
W*lnAT	0.520	-0.176	-0.157	-3.995	-1.711***	-0.992	-1.923***
W*lnPr	-0.168	-1.233	0.301***	-0.546	-0.713	-0.373*	1.311
W*lnWS	-0.470	-8.970	-0.245	0.672***	-0.112	-1.001*	4.814**
W*lnS	-3.961**	-2.968	-0.099	0.437	0.315	1.781	4.835***
W*lnEr	-0.007	-0.054*	-0.002	-0.015	0.001	-0.009	-0.115
W*lnES	-0.126	-0.712***	0.021	0.096	0.140	-0.006	0.094
W*lnER	0.041**	0.031*	0.024	0.012	0.039	0.017	0.026
R^2^	0.933	0.939	0.988	0.841	0.969	0.989	0.959
loglikehood	117.44	83.81	254.82	135.64	188.11	176.21	168.39

Note

* represents significance at the 10% level

** represents significance at the 5% level; and

*** represents significance at the 1% level.

#### 4.3.1 Comparative analysis of factors contributing to PM_2.5_ pollution

*(1) Effects of local meteorological factors on PM*_*2*.*5*_ *pollution in coastal urban agglomerations*. Meteorological factors significantly affected PM_2.5_ pollution in China's coastal urban agglomerations, and large spatial differences in the effects were observed. Wind speed and annual average temperature had negative effects on PM_2.5_ pollution, while there were significant positive and negative correlations between sunshine hours and average annual precipitation and PM_2.5_ pollution. Annual average wind speed has a significant inhibitory effect on PM_2.5_ concentration in the national-level urban agglomerations (Beijing–Tianjin–Hebei, Yangtze River Delta, and Pearl River Delta). The average annual temperature was highly correlated with latitude. The PM_2.5_ concentrations in the low-latitude Strait West Bank, Pearl River Delta, and Beibu Gulf urban agglomerations were significantly negatively affected by the average annual temperature, with the strongest effect observed in Beibu Gulf. When the annual average temperature increased by 1% in Beibu Gulf, the PM_2.5_ concentration increased by 5.79%. The sunshine hours inhibited PM_2.5_ pollution in Beijing, Tianjin, Hebei and central and southern Liaoning, but significantly increased the urban agglomeration in Beibu Gulf. This is because the northern urban agglomeration has longer sunshine hours than the southern urban agglomeration. With the increase of sunshine hours, the comprehensive effects of thermal convection, thermal and mechanical turbulence are strengthened, the surface inversion stratification is effectively destroyed, the vertical diffusion ability of the atmosphere is enhanced, which is conducive to the migration and diffusion of atmospheric pollutants. Therefore, the PM_2.5_ concentration in Beijing, Tianjin, Hebei and central and southern Liaoning urban agglomerations has been significantly affected. On the contrary, the sunshine duration of Beibu Gulf urban agglomeration is relatively short, which is not conducive to the diffusion of pollutants [44]. Precipitation significantly reduced PM_2.5_ pollution in the Pearl River Delta urban agglomeration but aggravated pollution in the Beijing–Tianjin–Hebei, Shandong Peninsula, and Strait West Bank urban agglomerations. In general, precipitation can wash PM_2.5_ out of the atmosphere, thereby reducing PM_2.5_ pollution [45]. However, the Beijing–Tianjin–Hebei and Shandong Peninsula urban agglomerations experiences short precipitation durations and low precipitation amounts along with serious pollution. After rain events, the static and stable meteorological conditions are not conducive to the spread of pollutants, and the precipitation actually increases PM_2.5_ pollution. The Strait West Bank urban agglomeration receives abundant precipitation, and the high humidity accelerates the accumulation of secondary particles, which in turn increases PM_2.5_ pollution.

In terms of spatial spillover effects, the temperature significantly reduced PM_2.5_ pollution in the Strait West Bank and Beibu Gulf urban agglomerations. Sunshine hours significantly reduced the concentration of PM_2.5_ in the urban agglomeration of central and southern Liaoning and the aggravated Beibuwan urban agglomeration. Precipitation reduced PM_2.5_ pollution in the Pearl River Delta urban agglomeration but aggravated pollution in the Shandong Peninsula urban agglomeration. In general, similar to wind speed, the more stable meteorological factors (temperature, sunshine, and precipitation) had the same effect on PM_2.5_ concentration in local and adjacent areas. The wind speed was regionally significant. Wind speed directly determines the pollutant diffusion distance and is an important factor affecting the spread of PM_2.5_ concentration. In this study, the influence of wind speeds different among the different urban agglomerations. Wind speed significantly aggravates PM_2.5_ pollution in Beijing-Tianjin-Hebei and Yangtze River Delta urban agglomerations, and reduces PM_2.5_ pollution in Pearl River Delta urban agglomerations. This is because the sources supplying pollution are mostly located upwind of the Beijing–Tianjin–Hebei and Yangtze River Delta urban agglomerations, and these areas have strong and persistent emissions. In contrast, the emissions that affect the Pearl River Delta urban agglomerations are generally small and are more likely to be carried away by the wind.

*(2) Effects of socioeconomic factors on PM*_*2*.*5*_ *pollution in coastal urban agglomerations*. Two direct effects, The proportion of tertiary industry and openness, have significantly reduced PM_2.5_ pollution in urban agglomerations in eastern China. In contrast, urbanization has significantly increased PM_2.5_ pollution. For the remaining socioeconomic factors, significant regional differences in their effects on PM_2.5_ pollution were observed in China's coastal urban agglomerations. Industrial agglomeration only had a positive effect on PM_2.5_ pollution in the Shandong Peninsula urban agglomeration; the opposite effect was observed in all other urban agglomerations. This is mainly because industrial agglomeration has a threshold effect on environmental pollution impact. When the level of industrial agglomeration was low, environmental pollution was aggravated; however, when industrial agglomeration reached a certain level, environmental pollution was reduced [46]. Currently, the high-energy-consuming industries in the Shandong Peninsula urban agglomeration are concentrated, but the level of agglomeration is low. The effects of technological innovation are phased. The Pearl River Delta urban agglomeration is the core area of technological innovation in China; the level of technology maturity in this area is high, resulting in reduced PM_2.5_ pollution [47]. However, technological innovation in the Strait West Bank urban agglomeration has only just begun, The introduction of external phase-out technology and the implementation of new technologies that are not yet mature can lead to the rapid development of intensive industry, causing a rebound effect in energy consumption and an increase in PM_2.5_ pollution [48]. Pollution of PM_2.5_ was aggravated. Population density significantly increased PM_2.5_ pollution in northern coastal urban agglomerations such as Beijing, Tianjin, Hebei, Shandong Peninsula and central and southern Liaoning, and reduced PM_2.5_ pollution in southern coastal urban agglomerations such as the Pearl River Delta, the West Bank of the Strait and Beibu Gulf, which was directly related to winter heating in northern China. Per capita GDP has greatly reduced PM_2.5_ pollution in Beijing-Tianjin-Hebei urban agglomeration, but it has aggravated PM_2.5_ pollution in Shandong Peninsula, Central-South Liaoning and West Bank of the Strait, of which central-South Liaoning and Beijing-Tianjin-Hebei are more affected. This is because in 2018, the tertiary industry of Beijing-Tianjin-Hebei urban agglomeration contributed more to per capital GDP, while the secondary industry dominated the urban agglomeration of central and southern Liaoning, and the high-polluting enterprises accounted for a larger proportion.

As a result of urban development foundations within urban agglomerations, the spatial spillover effects of socioeconomic factors are complex and show large regional differences. Specifically, a high degree of openness inhibited local PM_2.5_ pollution but increases the pollution in peripheral areas. This is primarily because foreign investment in large cities has promoted upgrades to local industries and accelerated the gradient transfer of backward production capacity to peripheral regions [49]. The level of urbanization was positively associated with local PM_2.5_ concentration but inhibited PM_2.5_ pollution in neighboring areas. Population density also inhibited PM_2.5_ pollution in peripheral areas. The central city with the largest population density attracts population from the surrounding area to the central city (i.e., the agglomeration effect); this has a mitigating effect on PM_2.5_ pollution in the surrounding areas [50]. Industrial agglomeration has increased PM_2.5_ pollution in the outer regions of the Yangtze River Delta and Shandong Peninsula urban agglomerations; in contrast, it suppressed PM_2.5_ pollution in the outer areas of the Pearl River Delta and Beibu Gulf urban agglomerations. The Central and South Liaoning, Strait West Bank, and Beibu Gulf urban agglomerations are growing and are in the stage of gradient transfer of backward production capacity to surrounding areas. The proportion of tertiary industry reduced the air quality in the surrounding areas. The Yangtze River Delta urban agglomeration is already in a mature stage, and this urban agglomeration, along with its peripheral areas, has formed a relatively stable functional division. The industrial structure continues to be optimized in this area, and The proportion of tertiary industry has a positive effect on air quality [51]. Scientific and technological innovation has reduced the spillover effect of PM_2.5_ pollution in the Beijing–Tianjin–Hebei and Pearl River Delta urban agglomerations. High-quality economic growth driven by the coordinated development of these urban agglomerations has also reduced pollution in neighboring areas. The increase in per capital GDP in the Shandong Peninsula urban agglomeration increased local PM_2.5_ pollution but improved the air quality in the adjacent areas. It shows that Shandong Province plays a prominent role as the undertaking grounding of industrial transfer in Beijing, Tianjin, Hebei.

*(3) Effects of pollution sources on PM*_*2*.*5*_ *pollution in coastal urban agglomerations*. Pollution sources play a significant role in PM_2.5_ pollution in China's coastal urban agglomerations. Building dust, vehicle exhaust, industrial energy consumption, and emissions directly contribute PM_2.5_ pollution in all of the urban agglomerations. Automobile exhaust was the main source of PM_2.5_ pollution in the Beijing–Tianjin–Hebei, Yangtze River Delta, Shandong Peninsula, and Beibu Gulf urban agglomerations. From 2010–2016, the number of motor vehicles in the above urban agglomerations increased by 49%, 47%, 30%, and 55%, respectively. It is much higher than other urban agglomerations along the coast of China. Building dust was the main source of PM_2.5_ concentration in the Pearl River Delta and the Central and Southern Liaoning urban agglomerations; the building dust is associated with the rapid urbanization in these areas.

Considering spatial spillover effects, the PM_2.5_ pollution sources in urban agglomerations had positive effects on PM_2.5_ concentration in the surrounding areas, and significant regional differences were observed. Automobile exhaust and industrial emissions had large effects on PM_2.5_ concentration in the areas surrounding the Beijing–Tianjin–Hebei and Strait West Bank urban agglomerations, primarily due to the recent rapid growth in vehicle ownership and industrial emissions in these two urban agglomerations. Building dust contributed significantly to PM_2.5_ concentration in the areas surrounding the Shandong Peninsula and the Central and Southern Liaoning urban agglomerations. Industrial energy consumption had a significant spatial spillover effect on the Yangtze River Delta urban agglomeration; the proportion of fossil energy use in the Yangtze River Delta was as high as 85% during the study period, and the energy consumption of the industrial sector accounted for 70% of the total energy consumption. Local PM_2.5_ pollution increased by 1% during the study period, while the concentration of PM_2.5_ in the adjacent area increased by 0.275%.

*(4) Effects of local ecological factors on PM*_*2*.*5*_ *pollution in coastal urban agglomerations*. Ecological factors had little effect on PM_2.5_ in China's coastal urban agglomerations. Cleanliness and ecological safety significantly reduced PM_2.5_ pollution, while ecological damage increased PM_2.5_ pollution in the coastal urban agglomerations. Cleanliness directly reduced the PM_2.5_ concentration in the Beijing–Tianjin–Hebei, Yangtze River Delta, and Pearl River Delta urban agglomerations, consistent with implementation of PM_2.5_ pollution prevention and control in key national regions. The effect of ecological safety degree on PM_2.5_ pollution was only significant in the Yangtze River Delta urban agglomeration, and the effect was small.

In terms of spatial spillover effects, cleanliness and ecological security had negative spatial spillover effects on PM_2.5_ pollution, while the opposite trend was observed for ecological damage. That is, cleanliness and ecological safety suppressed PM_2.5_ pollution in the areas surrounding the urban agglomerations, while ecological damage increased PM_2.5_ pollution in the surrounding areas.

#### 4.3.2 Analysis of the main factors affecting PM2.5 pollution

The factors with the five strongest positive and negative effects on PM_2.5_ pollution in China's coastal urban agglomerations are summarized in (Table 5). The factor with the largest coefficient (strongest effect) was defined as the main controlling factor. Among the types of factors, the contribution to decreases in PM_2.5_ pollution decreased from the following order: meteorological factors > socioeconomic factors > pollution source > ecological factors. Thus, meteorological factors were the core controlling factors contributing to reductions in PM_2.5_ pollution in China's coastal urban agglomerations, while socioeconomic factors also contributed greatly to the improvement in PM_2.5_ pollution.

**Table 5:** Rankings of the contributions of different factors to PM_2.5_ concentration in China's coastal urban agglomerations.

Urban Agglomeration	Central and South Liaoning	Beijing–Tianjin–Hebei	Shandong Peninsula	Yangtze River Delta	Strait West Bank	Pearl River Delta	Beibu Gulf
lnIa	5+		5-			3+	
Fo	3-						5-
lnPd		5-					4-
UR	1+				4+		
lnPGDP	4+				5+		
lnTE		4-		4+			
lnIEC				5+			
lnBd						4+	
lnAT		5+			1-	1-	1-
lnPr		3-	3+		1+	5-	
lnWS		1-		1-		2-	2-
lnS	1-	3+					
lnES				5-			
lnER						5+	
W*lnPM_2.5_	3+		1+	3-	2+	1+	
W*lnIa			5+	3+			
W*lnIha	2+			4-			
W*Ti		4+	1-		3+		5+
W*Fo		1-	2+	1+		2+	3+
W*lnPd	4-		2-	2-	4-	4-	4+
W*UR			4-		5-		
W*lnPGDP			3-				
W*lnTE		2-					
W*lnAT					2-		3-
W*lnPr			4+		3-		
W*lnWS				1+		3-	2+
W*lnS	2-	2+					1+

Note: + indicates a positive effect on PM_2.5_ pollution,—indicates has a negative effect on PM_2.5_ pollution. The colored bars indicate an effect on PM_2.5_ pollution, with the rankings (1–5) corresponding to the top five contributions (both positive and negative) for each urban agglomeration.

Among the different controlling factors, meteorological factors (average wind speed, sunshine hours, average annual temperature, and precipitation) had the most direct effects on PM_2.5_ pollution in China's coastal urban agglomerations, followed by socioeconomic factors (openness and urbanization). However, the main controlling factors differed among the urban agglomerations. Among the factors that improved air quality, annual average wind speed was the main controlling factor in the Beijing–Tianjin–Hebei and Yangtze River Delta urban agglomerations; sunshine hours was the main controlling factor in the central and southern Liaoning urban agglomeration, while technical support was the main controlling factor in the Shandong Peninsula urban agglomeration. Average annual temperature was the main controlling factor in the Strait West Bank, Pearl River Delta, and Beibu Gulf urban agglomeration. Among the factors that increased PM_2.5_ pollution, the main controlling factors in the Central and South Liaoning, Beijing–Tianjin–Hebei, and Yangtze River Delta urban agglomerations were socioeconomic factors. In the Beijing–Tianjin–Hebei and Yangtze River Delta urban agglomerations, the spatial spillover of external opening degree was the main controlling factor. The main controlling factor of urban agglomeration in central and southern Liaoning is urbanization rate. The spatial spillover effect contributed the most to the increase in PM_2.5_ pollution in the Shandong Peninsula and Pearl River Delta urban agglomerations. The PM_2.5_ concentration showed significant spatial mobility in the highly polluted urban agglomerations, indicating an urgent need for cross regional pollution prevention and control interventions. The main controlling factors in the Strait West Bank and Beibu Gulf urban agglomerations were meteorological elements, namely precipitation and sunshine hours (Table 6).

**Table 6:** Analysis of the main factors controlling PM_2.5_ pollution in China's coastal urban agglomerations.

Urban Agglomeration		Central and South Liaoning	Beijin–Tianjin–Hebei	Shandong Peninsula	Yangtze River Delta	Strait West Bank	Pearl River Delta	Beibu Gulf
Main controlling factors	Main factor contributing to the reduction in PM_2.5_ concentration	S	WS	Ti	WS	AT	AT	AT
	Main factor contributing to the increase in PM_2.5_ concentration	Ur	Fo	W*lnPM2.5	Fo	Pr	W*lnPM2.5	S

## 5 Results and discussion

### 5.1 Results

The rapid urbanization and industrialization of coastal urban agglomerations has resulted in serious pollution-related environmental problems, including severe smog. Coastal urban agglomerations have become highly polluted with PM_2.5_. Past studies have primarily examined single factors affecting PM_2.5_, including meteorological elements, industrial emissions, automobile exhaust, and urbanization. The temporal and spatial distribution characteristics of PM_2.5_ pollution in China's coastal urban agglomerations were analyzed, and the spatial Dubin model was applied to establish the effects of meteorological, pollution source, socioeconomic, and ecological factors on PM_2.5_ pollution. The main factors controlling PM_2.5_ pollution in China's coastal urban agglomerations were identified. The results can be summarized as follows.

1. From 2010–2016, the proportion of cities with low PM_2.5_ concentration in China's coastal urban agglomerations increased, and the overall PM_2.5_ concentration showed a downward trend. The air quality in China's coastal urban agglomerations improved overall. With the exception of the Yangtze River Delta urban agglomeration, the PM_2.5_ concentrations in the coastal urban agglomerations decreased. The year 2013 was an important turning point in PM_2.5_ concentration [6]. During the study period, the decreasing order of PM2.5 concentration was Shandong Peninsula > Beijing, Tianjin and Hebei > Yangtze River Delta > Central and Southern Liaoning > Pearl River Delta > Beibuwan > West Bank of the Strait.
2. From 2010–2016, the PM_2.5_ concentration exhibited significant spatial differences in the study area. PM_2.5_ concentration was significantly higher in the high-latitude areas compared to in the low-latitude areas. PM_2.5_ concentration also showed significant spatial differences within individual urban agglomerations, with higher concentrations observed in the northern parts or the urban agglomerations.
3. Significant spatial autocorrelation and spatial heterogeneity in PM_2.5_ pollution were observed in China's coastal urban agglomerations on the municipal scale. The northern coastal urban agglomerations formed a relatively stable and continuous high-pollution zone. In contrast, the southern coastal urban agglomerations formed a stable zone of good air quality. The spatial distribution of cold spots and hotspots in PM_2.5_ concentration changed significantly on the national level, while the changes were small on the regional level.
4. The spatial Dubin model was used to analyze the driving factors of PM_2.5_ pollution in coastal urban agglomerations. Spatial interactions had obvious effects of PM_2.5_ pollution, and the spatial spillover effect was significant. Meteorological, socioeconomic, pollution source, and ecological factors all affected the spatial characteristics of PM_2.5_ pollution during the study period. Among the different factors, meteorological factors had the most significant effects on PM_2.5_ concentration. Pollution source factors had significant positive effects on PM_2.5_ concentration. Socioeconomic and ecological factors had different effects, both positive and negative, on PM_2.5_ concentration in different urban agglomerations. The rapid increases in population density, automobile emissions, and urbanization along with inefficient industrial structure were important driving factors of PM_2.5_ pollution in China's coastal urban agglomerations.

### 5.2 Discussion

The rapid urbanization and industrialization of coastal urban agglomerations has resulted in serious pollution-related environmental problems, including severe smog. Coastal urban agglomerations have become highly polluted with PM_2.5_. Past studies have primarily examined single factors affecting PM_2.5_, including meteorological elements, industrial emissions, automobile exhaust, and urbanization. The results of this study indicated that many factors significantly affected PM_2.5_ pollution in China's coastal urban agglomerations from 2010–2016, with the overall effect being a mixed effect. Moreover, the main factors controlling PM_2.5_ pollution differed among the urban agglomerations. Among the different types of factors considered, the meteorological factors had the largest effects on PM_2.5_ concentration; however, meteorological elements are difficult to control, and the elastic coefficient of change is small. Thus, the socioeconomic factors, which can be controlled, should be considered when formulating air pollution prevention and control measures. Since the effects of different factors on PM_2.5_ pollution differed between the coastal urban agglomerations, it is critical to understand the mechanisms by which the different factors affect PM_2.5_ pollution in coastal urban agglomerations; this understanding is key to ensuring sustainable development and sound regional policy.

The spatial differences in PM_2.5_ pollution in China's coastal urban agglomerations result from various factors. The spatial distribution characteristics of PM_2.5_ concentration in China’s coastal urban agglomerations were evaluated in this study to develop a comprehensive understanding of PM_2.5_ pollution. From the perspective of spatial spillover, revealing the spatial conduction mechanism of PM_2.5_ pollution, it can clarify the spatial relationship between various elements and obtain the interaction mechanism of multiple elements of each urban agglomeration. Based on geographical spatial association characteristics, the findings of this study provide a multidimensional perspective that includes meteorology, ecology, and socioeconomic factors. However, because the formation of haze is extremely complicated, the research needs to be improved and deepened. The system of factors affecting PM_2.5_ concentration still needs to be improved to account for increasing agricultural production and biofuel combustion, for example. In the future, research on PM_2.5_ pollution should incorporate various disciplines, and multidimensional, multi temporal, and multidisciplinary research is needed to further clarify the causes, mechanisms, and flow paths of PM_2.5_ pollution. Future work will include field research on PM_2.5_ pollution in each region to provide a foundation for the development of regional policy.
